# LOX expression in primary nasopharyngeal carcinoma: correlation with prognostic parameters and outcome

**DOI:** 10.18632/oncotarget.6996

**Published:** 2016-01-23

**Authors:** Yi-Jun Hua, Hai-Yun Wang, Ling-Quan Tang, Qiu-Yan Chen, Jian-Yong Shao, Hai-Qiang Mai

**Affiliations:** ^1^ Sun Yat-sen University Cancer Center, State Key Laboratory of Oncology in South China, Collaborative Innovation Center for Cancer Medicine, Guangzhou 510060, China; ^2^ Department of Nasopharyngeal Carcinoma, Sun Yat-sen University Cancer Center, Guangzhou 510060, China; ^3^ Department of Molecular Diagnostics, Sun Yat-sen University Cancer Center, Guangzhou 510060, China

**Keywords:** nasopharyngeal carcinoma, lysyl oxidase (LOX), expression, survival, prognosis

## Abstract

Lysyl oxidase (LOX) is an extracellular matrix-remodeling enzyme that plays important roles in tumor development and progression. To evaluate the prognostic value of LOX levels in primary nasopharyngeal carcinoma, we performed an immunohistochemical analysis using 233 tissue biopsy specimens from as many patients. We found that the extent of immunohistochemical LOX staining correlated inversely with the clinicopathological features and survival. High LOX expression correlated with decreases in 5-year survival, overall survival, distant metastasis-free survival and disease-free survival (*p* < 0.05). Cox regression analysis confirmed that LOX was a significant prognostic indicator of increased risk of 5-year mortality for all patients (hazard ratio [HR], 1.670; 95% confidence interval [95% CI], 1.033–2.701 [*p* < .005]). Higher LOX expression was also an independent predictor of poor prognosis in patients with nasopharyngeal carcinoma. These findings suggest LOX may be a new biomarker predictive of NPC prognosis and may also be a useful treatment target.

## INTRODUCTION

Lysyl oxidase (LOX) is a copper-dependent amine oxidase and the most studied member of a five-protein family that includes LOX and four LOX-like proteins (LOXL1–4) [1]. LOX functions in part extracellularly, catalyzing the covalent cross-linkage of collagen and elastin fibers within the extracellular matrix. LOX also acts intracellularly to increase cell differentiation/motility, adhesion and migration, as well as gene transcription [2–5].

Aberrant LOX expression and activity has been observed in various cancerous tissues and neoplastic cell lines [6]. As a component of the extracellular matrix that enhances cell motility and migration, LOX could play a key role in tumor development and progression [7]. Several studies have shown that LOX transcription is increased in tumors of the breast, central nervous system, and head and neck, among others, as well as in various cancer cell lines [7–11]. However, the contribution made by LOX to the progression of nasopharyngeal carcinoma (NPC) has not been examined previously. In the present study, therefore, we assessed LOX expression in a cohort of patients with NPC to determine its association with survival.

## RESULTS

### Patient characteristics

Among the 233 patients studied, 173 (74.2%) were male, and the median age was 46.27 years (range, 17– 76 years). Thirty-nine tumors (16.7%) were diagnosed as non-keratinizing differentiated carcinoma (NKDC), 188 (80.7%) as non-keratinizing undifferentiated carcinoma (NKUC), and 5 (2.1%) as keratinizing squamous cell carcinoma (KSCC). Seventy patients (30.0%) were stage I or II, and 167 patients (70.0%) were stage III or IV. The median follow-up time was 71.60 months (range, 6–115 months). During the follow-up period, 69 (29.6%) patients died and 77 (33.0%) experienced disease progression. Of the 77 patients experiencing progression, 42 developed distant metastasis, 26 developed local-regional relapse, and 9 experienced both distant metastasis and relapse. The detailed clinical information is summarized in Table 1.

**Table 1:** Characteristics of nasopharyngeal carcinoma patients (*n* = 233)

Characteristic	NPC patients: n (%)
**Gender**	
Female	60 (25.8%)
Male	173 (74.2%)
**Age**	
Median (range)	46.27 (17–76)
Mean ± SD	46.10 (11.0)
**Clinical stage**	
I–II	70 (30.0%)
III–IV	163 (70.0%)
**T classification**	
T1 + T2	101 (43.3%)
T3 + T4	132 (56.7%)
**N classification**	
No	67 (28.8%)
N1 + N2 + N3	166 (71.2%)
**Progression**	
Yes	77 (33.0%)
No	156 (67.0%)
**Death**	
Yes	69 (29.6%)
No	164 (70.4%)
**WHO histological classification**	
NKUC	188 (80.7%)
NKDC	39 (16.7%)
KSCC	5 (2.1%)
Missing	1 (0.4%)

SD, standard deviation; WHO, World Health Organization; NKUC, nonkeratinizing undifferentiated carcinoma; NKDC, non-keratinizing differentiated carcinoma; KSCC, keratinizing squamous cell carcinoma.

### Different patterns of LOX expression in NPC

**Figure 1:**
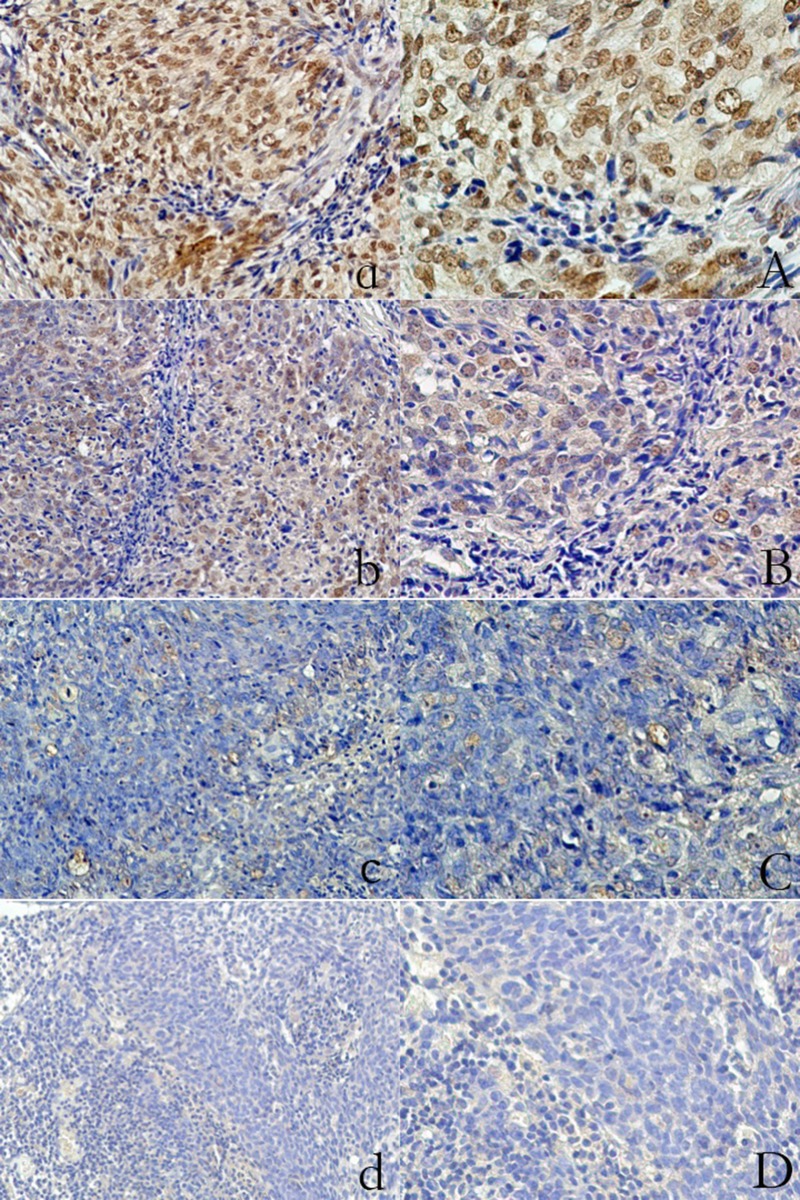
Expression of LOX in primary nasopharyngeal carcinoma (IHC staining) Strong expression (**a**) magnification, ×200; (**A**) magnification, ×400; Moderate expression: (**b**) magnification, ×200; (**B**) magnification, ×400; Weak expression: (**c**) magnification, ×200; (**C**) magnification, ×400; Negative: (**d**) magnification, ×200; (**D**) magnification, ×400.

LOX expression was assessed in all 233 NPC cases studied. The protein was localized predominantly in the cytoplasm and nucleus of tumor cells. High LOX expression was observed in 89 (38.20%) tumors. Of these, 21 cases had a score of 6, 38 cases had a score of 5 and 30 cases had a score of 4 (Figure 1). The associations between clinicopathological factors and LOX expression are summarized in Table 2.

**Table 2:** Correlation between expression of LOX and clinicopathological parameters of NPC

Clinicopathological parameters	Expression of LOX		*P* value
	Low expression (144)	High expression (89)	
Gender			0.759
Female	36	24	
Male	108	65	
Age			
< 46	74	39	0.282
≥ 46	70	50	
T classification			0.893
T1–2	63	38	
T3–4	81	51	
N classification			0.883
No	42	25	
N1–3	102	64	
Clinical stage			1.000
I–II	43	27	
III–IV	101	62	
WHO histological classification			0.132
NKUC	121	67	
NKDC	21	18	
KSCC	1	4	
Missing	1	0	

Survival analysis and prognostic significance of LOX expression

**Figure 2:**
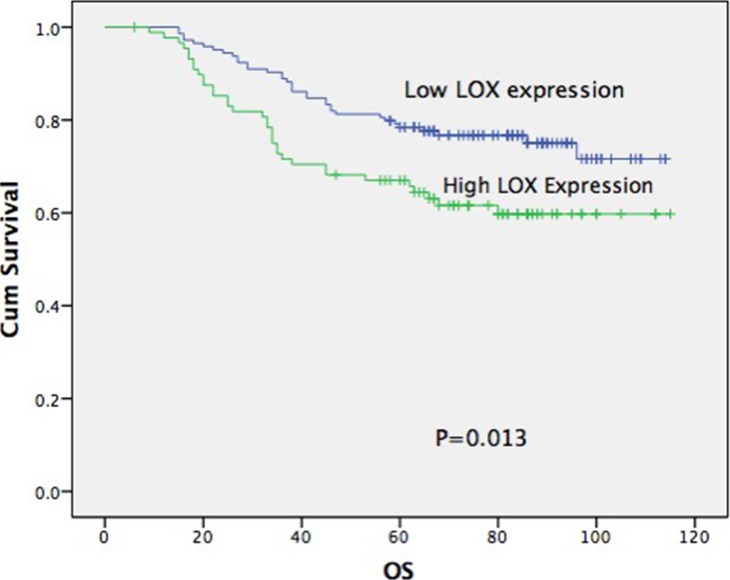

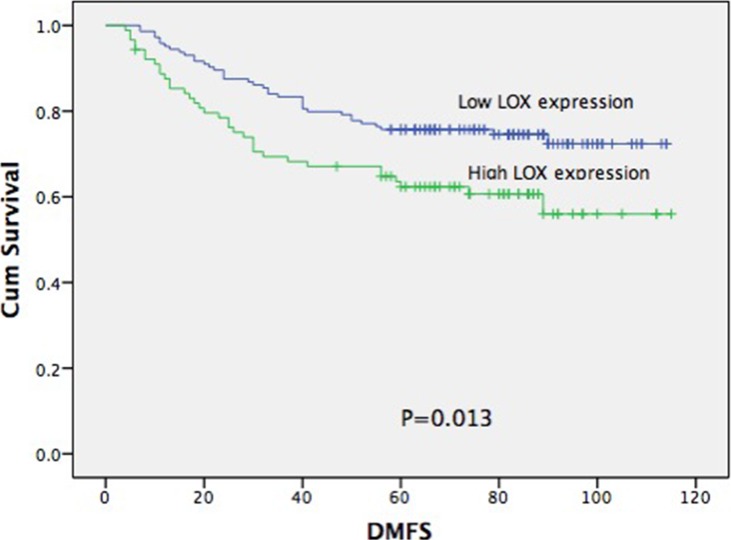

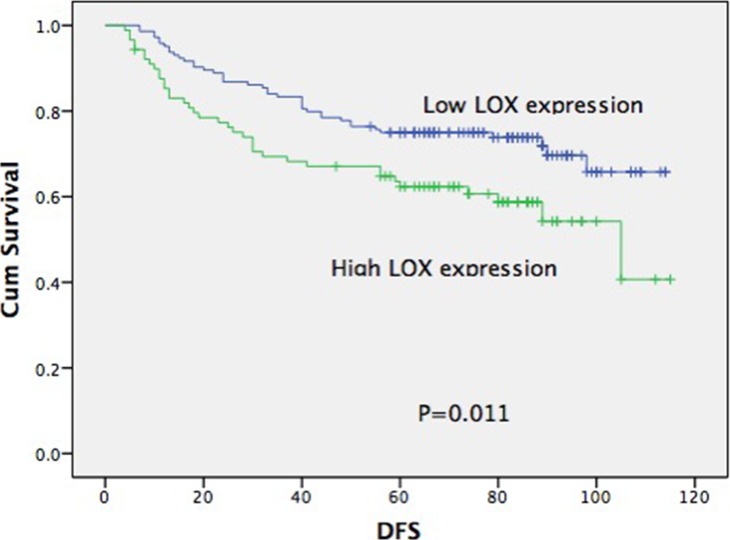
Overall survival curve, distant metastasis-free and disease-free survival curve according to LOX expression

The results showed that patients strongly expressing LOX had lower overall survival (61.44% vs. 76.88%, *p* = 0.011), distant metastasis-free survival (62.12% vs. 75.66%, *p* = 0.013) and disease-free survival (62.12% vs. 74.95%, *p* = 0.011) rates than those only weakly expressing low LOX Figure 2 (Table 3). Kaplan-Meier analysis also showed that age, T, N, clinical stage and LOX expression significantly affected survival (*p* < 0.05; Table 4). Cox multiple regression analysis showed that age, clinical stage and LOX expression are independent factors affecting survival (Table 4).

**Table 3:** Correlation of LOX expression with the prognosis of patients with NPC

5-Year rate	LOX expression		Chi square	*P* value
	high (%)	low (%)		
LRCR	93.77	95.37	1.204	0.273
DMFS	62.12	75.66	6.190	0.013
DFS	62.12	74.95	6.464	0.011
OS	61.44	76.68	6.543	0.011

**Table 4:** Univariate and multivariate Cox regression analyses of predictors of overall survival

Variable	R	Cl (95%)	P
Univariate analysis (*n* = 233)			
Age	2.200	1.325–3.652	0.002
Gender	0.641	0.351–1.172	0.149
T stage (T1–2/T3–4)	2.300	1.355–3.903	0.002
N stage (N0/N1–3)	2.414	1.267–4.601	0.007
Clinical stage (I–II/III–IV)	2.958	1.512–5.786	0.002
Lox (low/high)	1.825	1.138–2.927	0.013
WHO histological classification	1.075	0.643–1.797	0.784
Multivariate analysis (*n* = 233)			
Age	2.115	1.271–3.519	0.004
Clinical stage (I–II/III–IV)	1.783	1.108–2.868	0.017
Lox (low/high)	3.159	1.612–6.191	0.001

HR, hazard ratio; 95% CI, 95% confidence interval; LOX, lysyl oxidase.

## DISCUSSION

NPC is a common malignancy in southern China. Radiotherapy has proven to be an effective treatment of both primary and recurrent NPC, and the TNM stage is still the most frequently used tool for determining the prognosis of patients with NPC. This classification system takes into account the extension of tumor invasion (T), the involvement of lymph nodes (N) and the presence of distant metastases (M). In the present study, univariate analysis showed that age, extent of invasion, involvement of lymph nodes and TNM stage are all associated with prognosis, which was consistent with the results of earlier studies [12, 13].

Our focus was on the role of LOX expression in NPC. We showed that high LOX expression was significantly associated with a low 5-year survival rate and low disease-free survival. We further demonstrated that LOX is an independent prognostic factor in primary NPC. These results are consistent with other studies of head and neck cancers and oral cancer. They are also consistent with a study of oropharyngeal squamous cell carcinoma (OSCC), in which LOX expression was shown to be an independent prognostic biomarker predictive of lymph node metastasis [2].

As they grow, solid tumors establish a pathophysiologic microenvironment characterized by an irregular microvascular network and regions of hypoxia [14]. LOX staining was mainly in the cytoplasm and nucleus of NPC cells. It was previously shown that extracellular LOX is able to enter the cytosol of smooth muscle cells and then concentrate in the nucleus of through an unknown mechanism [15]. The heterogeneous distributions of LOX staining we observed in some specimens may reflect the effect of hypoxia, whereas the more homogeneous distribution is likely due to regulatory factors other than hypoxia. LOX reportedly modifies the tumor microenvironment to enhance cancer invasion and metastasis [16, 17]. As a copper-dependent amine oxidase, LOX catalyzes collagen and elastin cross-linking within the extracellular matrix [3]. This stabilizes these fibrous proteins and increases the stiffness of the matrix, which, in the microenvironment of tumors, was recently recognized to be an important determinant of tumor latency and malignancy [6, 18]. This is particularly noteworthy as LOX may also act intracellularly to enhance cell motility and migration [3, 6, 19, 20].

High LOX expression is reportedly associated with an increased recurrence rate and decreased overall survival in breast cancer, prostatic cancer, renal cell cancer, head and neck squamous cell cancer and gastric cancer [2, 9, 18]. In breast cancer, LOX appears to facilitate cancer cell migration and adhesion through hydrogen peroxide-mediated regulation of FAK/Src signaling [8, 21]. We also found that NPC patients with high LOX expression had a poorer prognosis as indicated lower rates of overall survival, distant metastasis-free survival and disease-free survival. Notably, however, there was no significant correlation between LOX expression and local-regional recurrence. Still, these results suggest that LOX could potentially serve clinically as one of several biomarkers of NPC prognosis.

There is some controversy concerning the biological relevance of LOX to the molecular pathogenesis of invasive carcinomas. Consistent with LOX playing an important role is the finding that its expression is increased in several types of malignant tumors as well as various cancer cell lines [7–11]. On the other hand, LOX appears to act as a tumor suppressor in human osteosarcoma and NPC cell lines [22, 23], and decreased LOX activity was noted in fibrosarcoma, rhabdomyosarcoma and choriocarcinoma cell lines [20]. The reasons for the contradictory results obtained for LOX in various cancers is likely multifactorial. For example, changes in gene expression patterns observed *in vitro* must be interpreted with caution when applied *in vivo*. In addition, LOX expression may vary during the different stages of transformation as the molecular environment of the tumor changes in the different tissue types [2].

In conclusion, the results of our study suggest that high LOX expression is associated with tumor progression and a poor prognosis in patients with primary NPC. They also suggest LOX may be a new biomarker predictive of NPC prognosis and may also be a useful treatment target.

## MATERIALS AND METHODS

Tissue specimens were obtained from pathological biopsies of 233 consecutive patients with primary NPC at Sun Yat-sen University Cancer Center between 2000 and 2001. The specimens were collected according to a protocol approved by the Research Medical Ethics Committee of Sun Yat-Sen University Cancer Center. The cases were selected based on the following criteria: pathologically confirmed diagnosis of NPC with biopsy specimens available for tissue microarray (TMA) construction; no previous malignant disease or second primary tumor; and no history of radiotherapy, chemotherapy or surgical treatment. The pathologic stage and linear extent of invasion for each specimen were determined by a panel of three pathologists. The patient characteristics are shown in Table 1. Tumors were restaged using the seventh edition of the American Joint Committee on Cancer Staging manual. All patients were treated with standard curative radiotherapy with or without chemotherapy. All patients underwent radiotherapy using a two-dimensional technique. The accumulated radiation dose to the primary tumor was 68–70 Gy. The metastatic lymph node-positive and lymph node-negative neck tissues received radiotherapy to a total dose of 60–62 Gy and 50 Gy, respectively. The follow-up period was defined as the time from diagnosis to the date of death or to the time of censure if the patient was still alive.

Disease progression was defined as death, progressive disease after primary treatment or recurrence (local progression) and/or the development of new distant metastases (distant progression).

### Tissue microarray construction

Tumor tissues were fixed in formalin and embedded in paraffin in a tissue microarray (TMA) conformation (Beecher Instruments, Silver Spring, MD). Two cylindrical cores with diameters of 1.0 mm were removed from each donor paraffin block and transferred to premolded recipient paraffin blocks at defined array positions. Recipient paraffin blocks contained holes of appropriate size in a grid pattern that was maximally 11 holes wide by 14 holes long, allowing for 154 tissue cores per block. This construction design permitted multiple blocks with identical array patterns to be constructed simultaneously, serially sectioned at 5 μm, placed onto “charged” glass slides, and stored at 4°C.

### Immunohistochemistry (IHC) and evaluation

IHC was performed to examine LOX expression in NPC tissues using a primary LOX antibody (NB100– 2530) (1:200 dilution; Novus Biologicals, USA). The IHC results were evaluated and scored independently by three pathologists without knowledge of the patients' clinicopathological outcomes. A semi-quantitative estimate was made using a composite score obtained by adding the intensity of the staining and the relative abundance of positive cells. Staining intensity was graded as 0 (negative), 1 (weakly positive), 2 (moderately positive) or 3 (strongly positive). The abundance of positive cells was graded from 0 to 3 (0, no positive cells; 1, 1–10%; 2, 11–50%; 3, 51– 100%). The two individual parameters were added, resulting in an immunoreactivity score (IRS) ranging from 0 to 6.

The immunohistochemical cut-off for high expression of LOX was determined through receiver operating characteristic (ROC) curve analysis. The sensitivity and specificity for discriminating death and survival was plotted for each IHC score, thereby generating a ROC curve. The cut-off value was established as the point on the ROC curve where the sum of the sensitivity and specificity was maximized. We defined cases with IRS ≥ 4 as high expression and cases with IRS < 4 as low expression.

For the Kaplan-Meier survival analysis, a composite score greater than the median value was considered high expression, and a composite score less than or equal to the median value was considered low expression.

### Statistical analysis

Statistical analysis was performed using SPSS 18.0 statistical software (Chicago, IL, USA). The last follow-up date was 20 April 2012. The locoregional control rate (LRCR), disease-free survival (DFS), distant metastasis-free survival (DMFS) and overall survival (OS) were calculated using the Kaplan-Meier method. Durations were calculated from the date of treatment to the date of event occurrence or date of last follow-up. The χ^2^ test was used to evaluate the relationship between LOX expression and clinical and pathological features. Univariate correlates were determined using the log-rank test. Multivariate analysis was performed using the Cox proportional hazards model. Values of *p* < 0.05 were considered statistically significant.
